# Stick–slip boundary friction mode as a second-order phase transition with an inhomogeneous distribution of elastic stress in the contact area

**DOI:** 10.3762/bjnano.8.189

**Published:** 2017-09-08

**Authors:** Iakov A Lyashenko, Vadym N Borysiuk, Valentin L Popov

**Affiliations:** 1 Technische Universität Berlin, 10623 Berlin, Germany; 2 Sumy State University, 40007 Sumy, Ukraine; 3 National Research Tomsk State University, 634050 Tomsk, Russia,; 4 National Research Tomsk Polytechnic University, 634050 Tomsk, Russia

**Keywords:** boundary friction, dimensionality reduction, numerical simulation, shear stress and strain, stick–slip motion, tribology

## Abstract

This article presents an investigation of the dynamical contact between two atomically flat surfaces separated by an ultrathin lubricant film. Using a thermodynamic approach we describe the second-order phase transition between two structural states of the lubricant which leads to the stick–slip mode of boundary friction. An analytical description and numerical simulation with radial distributions of the order parameter, stress and strain were performed to investigate the spatial inhomogeneity. It is shown that in the case when the driving device is connected to the upper part of the friction block through an elastic spring, the frequency of the melting/solidification phase transitions increases with time.

## Introduction

The boundary friction mode occurs in tribological systems when the thickness of the lubricant layer separating two contacting surfaces is significantly smaller than the typical size of the surface roughness. At such a system configuration, the lateral motion of the friction surface is followed by a contact interaction between the asperities. A specific case of boundary friction is friction between two atomically flat surfaces separated by a layer of lubricant with thickness of a few atomic diameters [1–2], or even monolayers [3]. Such type of friction mode plays an important role in applied mechanics as it often occurs in nanometer-sized tribological systems that are commonly used in aerospace technologies, computer memory devices and electronic positioning systems [4]. Various experimental research has shown that in the boundary friction mode, the lubricant can undergo periodic phase transitions between the structure states which may lead to the stick–slip motion with non-monotonic time dependence of the friction force [1–2,4–5]. Stick–slip motion is known to cause fast destruction of the contact parts of microscopic devices, which is why it receives significant attention from the scientists and engineers.

The boundary friction mode can be described within the framework of several theoretical models [6–12] where lubricant melting is described either as a first-order [8–9], or a second-order [10–11] phase transition. It is worth mentioning that in three-dimensional systems, melting always appears as a first-order phase transition [13], while in the systems with confined lubricant, second-order phase transitions were observed in both numerical [14–16] and theoretical [10] studies. Moreover, recent experimental investigations [5] have shown that melting as a first-order phase transition is not possible for boundary lubricants consisting of spherically shaped molecules. However for the polymeric lubricant materials, first-order phase transition may occur [17].

In our previous work [18] we studied the stick–slip boundary friction mode considering lubricant melting as both first and second-order phase transitions with an inhomogeneous distribution of elastic stress in a contact area. This obtained results have shown that the melting begins at the edge of a contact area and propagates to its center, and the wave of melting is followed by a wave of recrystallization. Such inhomogeneous behavior was also observed in experiments [19–20]. However, in [18] we consider the motion of the friction surfaces with constant relative velocity, while in the real experiments, the driving device is applied to the upper surface through the elastic spring [1,4,6]. In such an experimental configuration, the velocities of the friction surface and driving device are not equal, which significantly affects the friction mode. In the present paper, we study this situation using a previously developed technique [18]. In our research we use a thermodynamic approach, as proposed in [10], which gives relevant physical results. The dependence of the order parameter on elastic strain in the lubricant layer, obtained using the above-mentioned thermodynamic approach, agrees with the similar data obtained from computational studies [14–16]. Moreover, strain–stress curves obtained in [10] are confirmed by experimental data [21].

## Results and Discussion

We consider a simplified case where the properties of the lubricant are independent of pressure and its behavior can be described within a thermodynamic approach [10]. Assuming that the melting of the lubricant develops as a second-order phase transition (which follows from the computer simulations [14–15] and experimental investigations [5]), the free-energy density can be written in the form [10]:

[1]
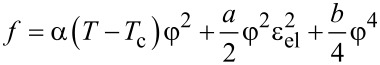


where *T* is the temperature of the lubricant; *T*_c_ is the critical temperature; ε_el_ is the shear component of the elastic stress; α, *a* and *b* are positive constants. The order parameter φ is a periodic component of the microscopic density of the material: in a solid-like state of the lubricant φ > 0, while in a liquid-like state φ = 0.

Using Equation 1 and the definition τ = ∂*f* / ∂ε_el_ [10,22] shear stresses that appear in the lubricant can be written in the form:

[2]
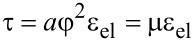


where we have introduced the shear modulus of the lubricant μ, that takes nonzero values only in solid-like states. The stationary values of the order parameter φ_0_ can be estimated from the condition ∂*f* / ∂φ = 0 in the following form:

[3]
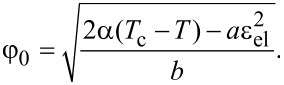


According to Equation 3, the stationary value of the order parameter φ_0_ decreases with the growth of both temperature *T* and elastic strain ε_el_. When the strain exceeds a critical value

[4]
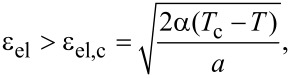


stationary values of the order parameter φ_0_ and shear modulus μ_0_ (according to Equation 2) equal to zero and the lubricant melts. In the case ε_el_ < ε_el,c_ as defined by Equation 4, the stationary stress in the lubricant can be expressed as

[5]
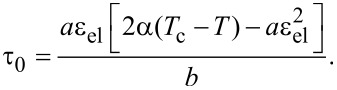


Equation 5 describes the strain–stress curve defined by the expansion parameters. However, it is more convenient to use experimentally observable values of the maximum stress τ^max^ and strain 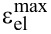. The relation between the expansion parameters α, *a* and *b* and values of τ^max^ and 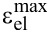 can be estimated from Equation 5 in the following form [18]:

[6]
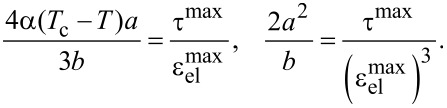


To study the kinetics of the lubricant we employed the Ginzburg–Landau–Khalatnikov evolutionary equation for the order parameter in the form:

[7]
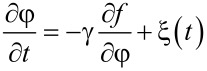


where γ is the kinetic parameter that defines the inertial properties of the system, ξ(*t*) represents random processes in the heat fluctuation of a small amplitude which cannot significantly affect the system behavior. Nevertheless, it is necessary to take them into account due to the peculiarities of the further numerical calculations [23]. In explicit form, Equation 7 can be written as

[8]
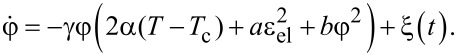


**Figure 1:**
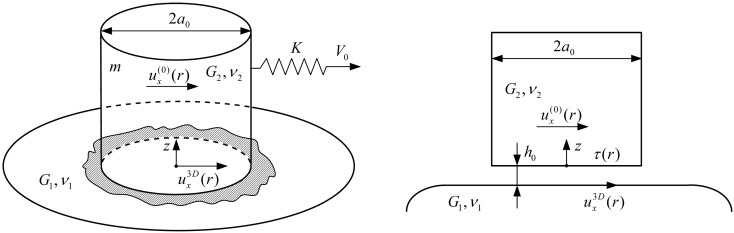
Geometrical scheme of the system under investigation. Stamp of a cylindrical shape with radius *a*_0_, made of material with shear modulus of *G*_2_ and Poisson ratio *v*_2_, placed over the material with elastic parameters *G*_1_,*v*_1_. Upper and lower friction blocks are separated by a layer of lubricant with thickness *h*_0_.

The thermodynamic approach described above can be used to investigate the boundary friction mode with different geometrical shapes of the contact area. In the present work we consider a tribological system as shown in Figure 1.

As can be seen from Figure 1, a cylindrically shaped flat-ended stamp with radius *a*_0_ is in contact with a lower surface through the lubricant layer with thickness *h*_0_. The materials of the top and bottom surfaces have the shear moduli *G*_1_,*G*_2_ and Poisson ratios *v*_1_,*v*_2_, respectively. This configuration can be reduced to the contact of a rigid stamp with half-space characterized by an effective shear modulus [24]

[9]
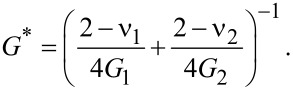


Assuming that the upper stamp has mass *m*, and the coordinate of the stamp center is *X*, let us consider the situation where the stamp is driven by a spring with the constant stiffness *K*. The free end of the spring moves with a constant velocity *V*_0_. Thus, the equation of motion for the upper friction block with mass *m* has the following form [4]:

[10]
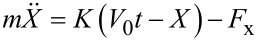


where *t* is the time, and *F*_x_ is the friction force between two contacting surfaces. The magnitude of the friction force *F*_x_ depends on the properties of the system shown in Figure 1.

As the upper stamp moves, local displacements of its surface in the contact area with a lubricant are defined by a radial distribution 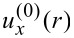, where *r* is the radial coordinate. Denoting the corresponding local displacements of a bottom surface as 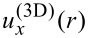, we can define the local shear strain in the lubricant layer as a function of the radial coordinate *r*:

[11]
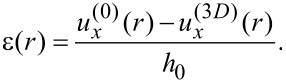


Knowing the distribution of strain ε(*r*) and order parameter φ(*r*) we can obtain the distribution of the stress in the lubricant according to Equation 2:

[12]
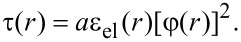


The distribution of the displacements 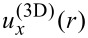 in Equation 11 is defined by shear stress. In our further investigations we will use the method of dimensionality reduction (MDR) [24–26], which allows us to reduce the three-dimensional problem (with general coordinate *r*) to an equivalent one-dimensional (coordinate *x*) with a possible reverse transition.

Within the MDR technique, a one-dimensional distribution of the force density can be defined from the known distribution τ(*r*) as:

[13]
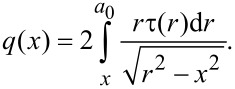


From the obtained *q*(*x*), the one-dimensional distribution of the displacements 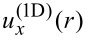 can be calculated as:

[14]
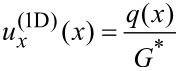


and the distribution 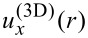 can be obtained from the equation:

[15]
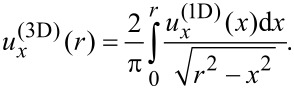


The elastic component of the friction force in the system can be defined in two ways (in one-dimensional and three-dimensional interpretations):

[16]
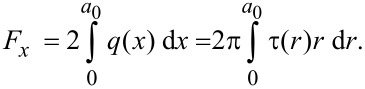


The aim of the present work is to take into account the elastic properties of the contacting materials in simulation of the kinetics of the boundary friction in the system shown in Figure 1. Let us introduce the brief algorithm of the simulation scheme. First, we need to set the initial distributions 
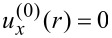
 and 
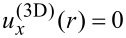
. After that, the procedure described in Equation 11–Equation 15 is repeated in loops and for every value of ε(*r*) a related value of the order parameter is calculated from Equation 8.

The displacement of upper stamp *X* can be estimated from the numerical solution of Equation 10 with friction force *F*_x_ calculated from Equation 16. With incremental growth of the upper friction block, coordinate *X* values of the distribution 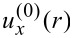 are also incremented by the same magnitude. Thus, at the beginning of motion, 
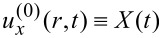
. However, 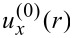 is set to zero when the lubricant melts (in numerical scheme 
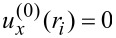
 when φ(*r_i_*) < 0.01 [18]).

In numerical calculations integrals of Equation 13, Equation 15 and Equation 16 were replaced by corresponding sums, while coordinates *x* and *r* were divided into *N* segments. All calculated distributions depending on radius *r* (or coordinate *x*), were computed at the points *r_i_* = *ia*_0_ / *N* (*x_i_* = *ia*_0_ / *N*), where 
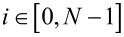
. In our simulation we set the time step to be Δ*t* = 10^−8^ s and number of segments *N* = 2000.

**Figure 2:**
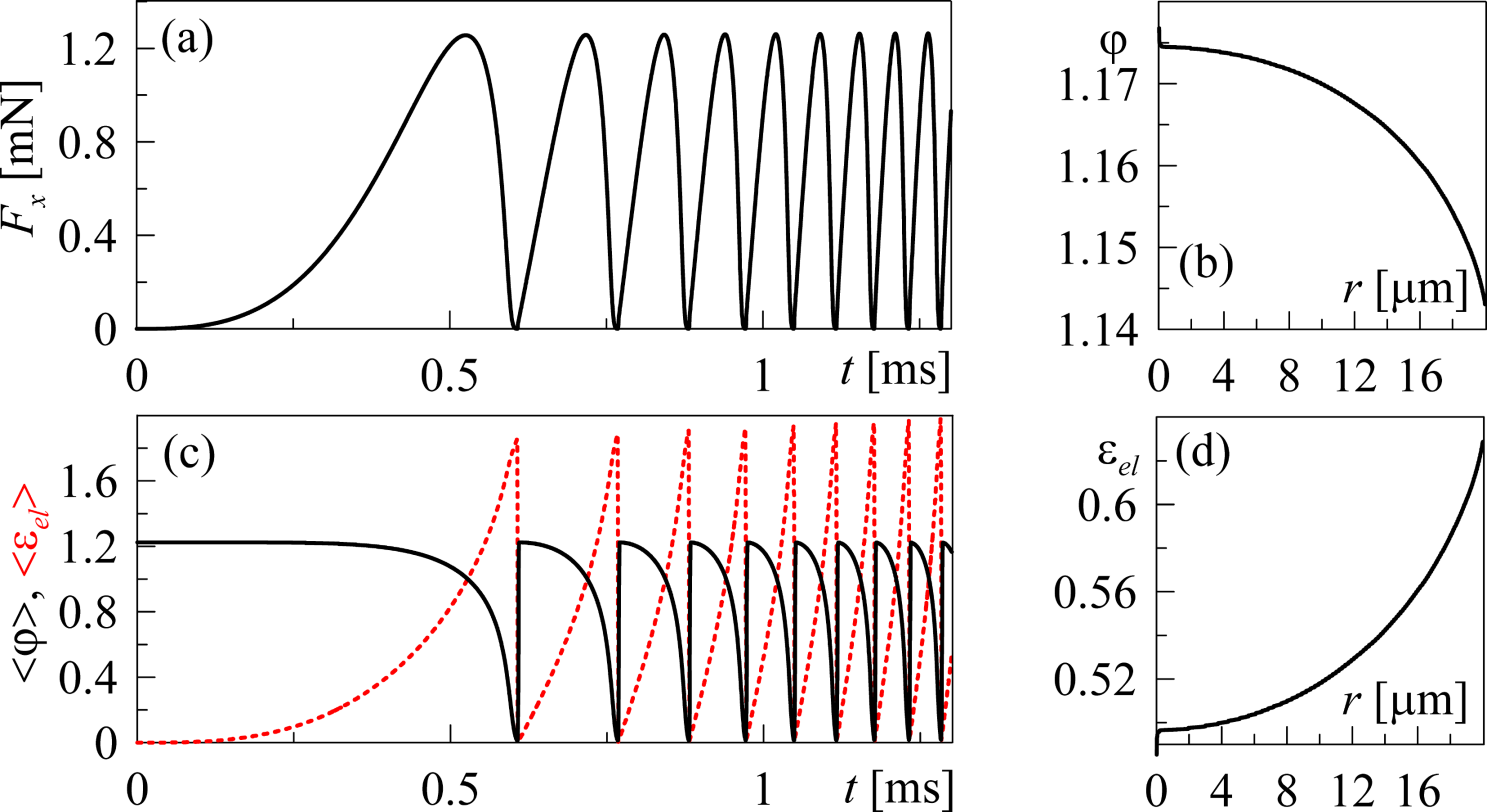
(a) Kinetic dependence of the friction force *F*_x_(*t*), calculated at parameters τ^max^ = 10^6^ Pa, 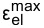 = 1.0, *a* = 10^6^ Pa, *h*_0_ = 10^−7^ m, γ = 10 (Pa·s)^−1^, *a*_0_ = 2·10^−5^ m, G* = 10^9^ Pa, *K* = 500 N/m, *m* = 0.1 kg, *V*_0_ = 1 m/s. (b) Spatial distribution of the order parameter φ(*r*) in the moment of time *t* = 1.3 ms, related to final point of the dependence shown in Figure 2a. (c) Time dependence of the mean values of the order parameter <φ> (solid line) and elastic strain <ε_el_> (dashed line). (d) Spatial distribution of the elastic strain ε_el_(*r*) at *t* = 1.3 ms.

Figure 2 shows the results of a numerical simulation of the shifting of the free end of the spring with constant velocity *V*_0_ at constant system parameters.

An analogous dependence was described in [18], where the motion of a stamp with constant velocity was considered. Such configuration relates to the case where the spring, shown in Figure 1, is replaced by the rigid coupler. However, in real experiments, the spring (finite stiffness) between the stamp and the driving device always exists.

The dependence shown in Figure 2 allows us to conclude that the stick–slip mode, with increasing frequency of the melting/solidification phase transitions, is established in the system. The growth of the frequency is caused by the increasing tension of the driving spring Δ*X* = *V*_0_*t* − *X* and the elastic force *F_u_* = *K*(*V*_0_*t* − *X*). The shear velocity of the upper stamp 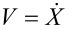 also increases according to Equation 10, while the time interval, during which the elastic stress ε_el_ exceeds the critical value, is reduced.

As it is follows from Figure 2b,d, melting of the lubricant occurs at the edge of the contact area and propagates to the center; this situation was observed earlier in theoretical [18,27] and experimental [19] research. It is worth mentioning that before the first melting, the dependencies *F*_x_(*t*), <φ>(*t*) and <ε_el_>(*t*) show the transition mode where monotonic growth of the friction force *F*_x_ and mean value of elastic strain <ε_el_> as well as <φ> are significantly slower. The corresponding time interval from the beginning of motion to the first melting is the largest due to the presence of the spring between the stamp and driving device [4]. Such a transition mode was not observed in [18] as the stamp was moving with constant velocity *V* from the very beginning of motion.

**Figure 3:**
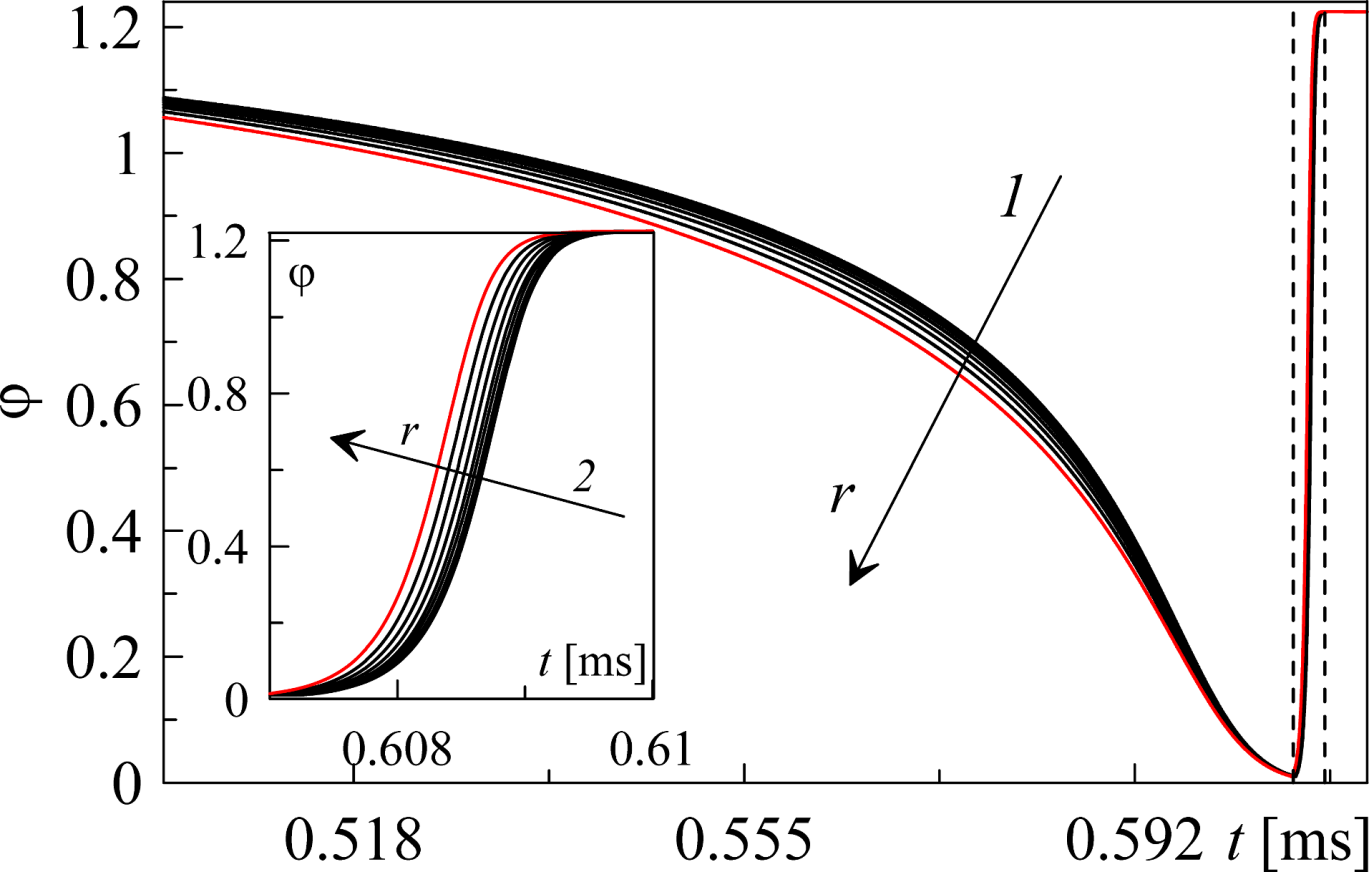
(a) Time dependence of the order parameter φ(*t*), calculated using the same parameters as in Figure 2 and corresponding to the melting process (arrow 1) before the first dashed line and to the recrystallization process (arrow 2) after the first dashed line for different values of the radial coordinate *r*. Arrows show the increment of a radial coordinate *r* from 2 to 18 μm, with a step of 2 μm. Inset shows the time interval between two dashed lines.

Additional time dependence of the order parameter φ(*t*) obtained for different values of the radial coordinate *r* is shown in Figure 3.

As it can be seen from Figure 3, melting begins at the edge of the contact area and is immediately followed by recrystallization. We also conclude that the inhomogeneous distribution of the parameters weakly affects the behavior of the considered tribological system in contrast to the case of a first-order phase transition, where the influence of inhomogeneity is significantly stronger [18].

The developed theoretical model of the boundary friction allows investigating of the influence of the temperature of the lubricant on the melting process. It is worth mentioning that the influence of the temperature was studied in a previous work [18] for the case where the stamp was moving with a constant velocity, and here we will discuss an analogous investigation for the system with the spring. As the coefficient

**Figure 4:**
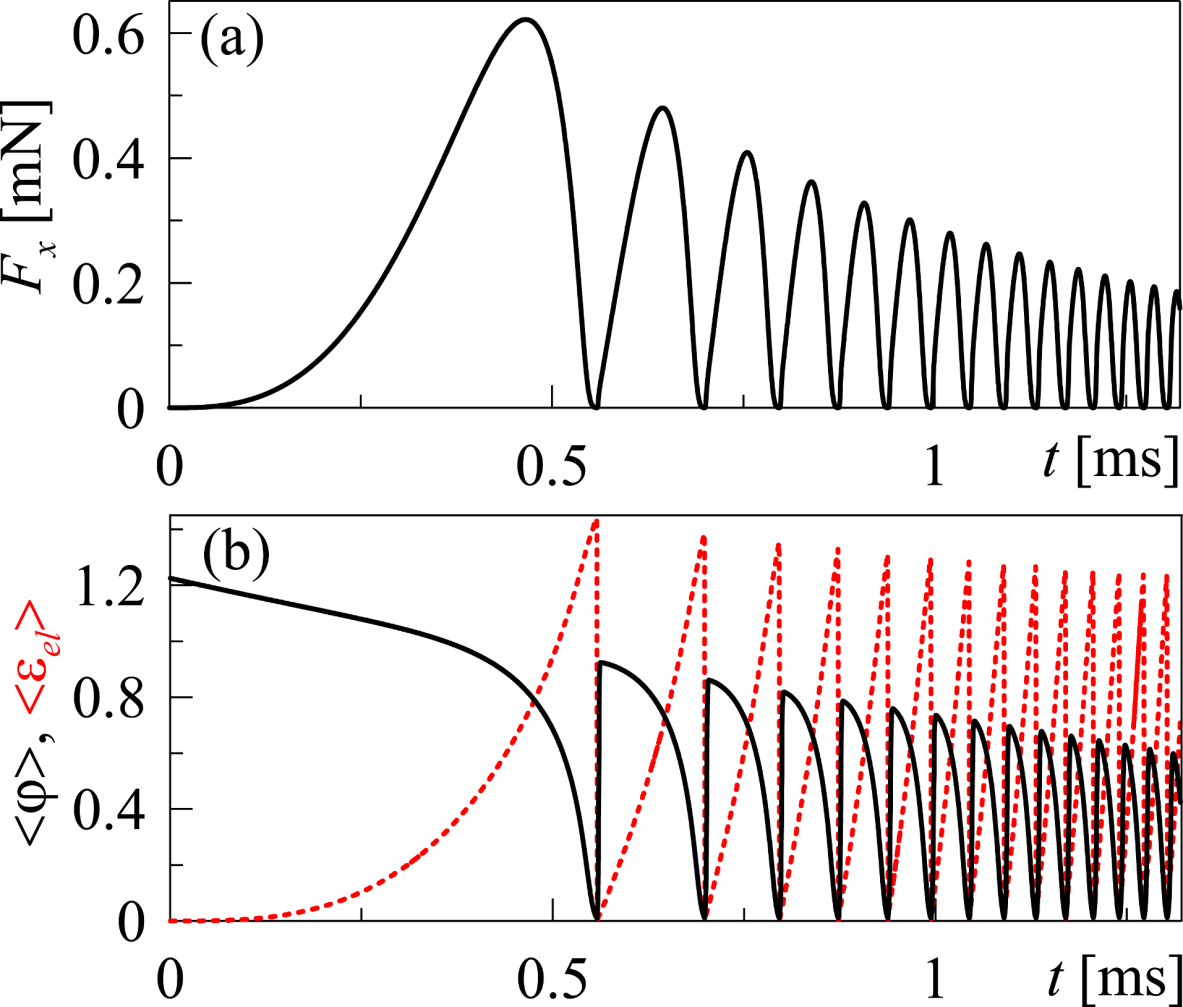
(a) Time dependence of the friction force *F*_x_ (Equation 16) using the parameters of Figure 2 and an increasing temperature according to Equation 18 using the parameters *A*_0_ = 1.5·10^6^ Pa and 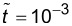
s. (b) Mean values of the order parameter <φ> and elastic strain <ε_el_> are according to the parameters of Figure 4a.

[17]
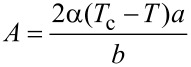


is the only parameter in the model that depends on the temperature *T*, the variations of this coefficient can be considered as variations of the temperature of the lubricant. As it follows from the definition, the coefficient *A* decreases with temperature increase. The dependence in Figure 2 is obtained using the parameters τ^max^ = 10^6^ Pa and 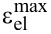 = 1.0, which corresponds to the value of *A* = 1.5·10^6^ Pa according to Equation 6. Figure 4 shows the time dependence of the friction force with monotonically decreasing coefficient *A* according to the relaxation law

[18]
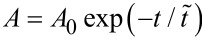


where *A*_0_ is the initial value of coefficient *A* at time *t* = 0, while 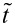 is the relaxation time. Equation 18 relates to the increase of the lubricant temperature.

The temperature of the lubricant can vary during the natural heat exchange with the environment (friction surfaces are considered as a thermostat) [28]. As it follows from Figure 4, the higher temperature of the lubricant leads to the reduced amplitude of the friction force, elastic stress and order parameter, which was previously observed in [18]. However, in the considered case, the frequency of the phase transitions increase with time due to the presence of the spring, as shown in Figure 1. Complete melting of the lubricant occurs at *A* = 2α(*T*_c_−*T*)*a* / *b* ≤ 0 (not shown in the figure) and is followed by a sliding mode with zero friction force *F*_x_ = 0 (only the elastic component of the friction force is considered within the proposed model).

Let us note that all presented dependencies relate to the particular situation where elastic stress increases according to Equation 11. However, in various experimental and theoretical studies, the boundary friction mode develops through an alternative mechanism where elastic stress, which cause the melting of a lubricant, can also exist in a liquid-like state [4,9,23]. At these conditions of time dependence, the friction force has a saw-like form and melting of the lubricant occurs when the shear velocity *V* exceeds some critical value. After the lubricant is melted, the elastic friction force becomes equal to zero, i.e. *F*_x_ = 0.

In the proposed model, we consider a quasi-static case where the elastic strain is defined by the displacement of the friction surfaces (see Equation 11) instead of the shear velocity *V*. Moreover, in the case of quasi-static contact, the viscous friction force is not considered, while in the standard dynamic model, it plays significant role [9]. Note however, that in most cases, the boundary lubricant layers will exhibit non-Newtonian behavior, so obtaining the dependence of viscous friction force on shear velocity may represent another difficult challenge [29]. Thus, in our model, the increase in the shear velocity *V* causes the increase in the frequency of phase transitions, and a critical value of *V* related to the complete melting of the lubricant is not observed. However, it is worth mentioning that the developed approach allows us to investigate the physical processes directly in the contact area, which is not possible within standard models.

**Figure 5:**
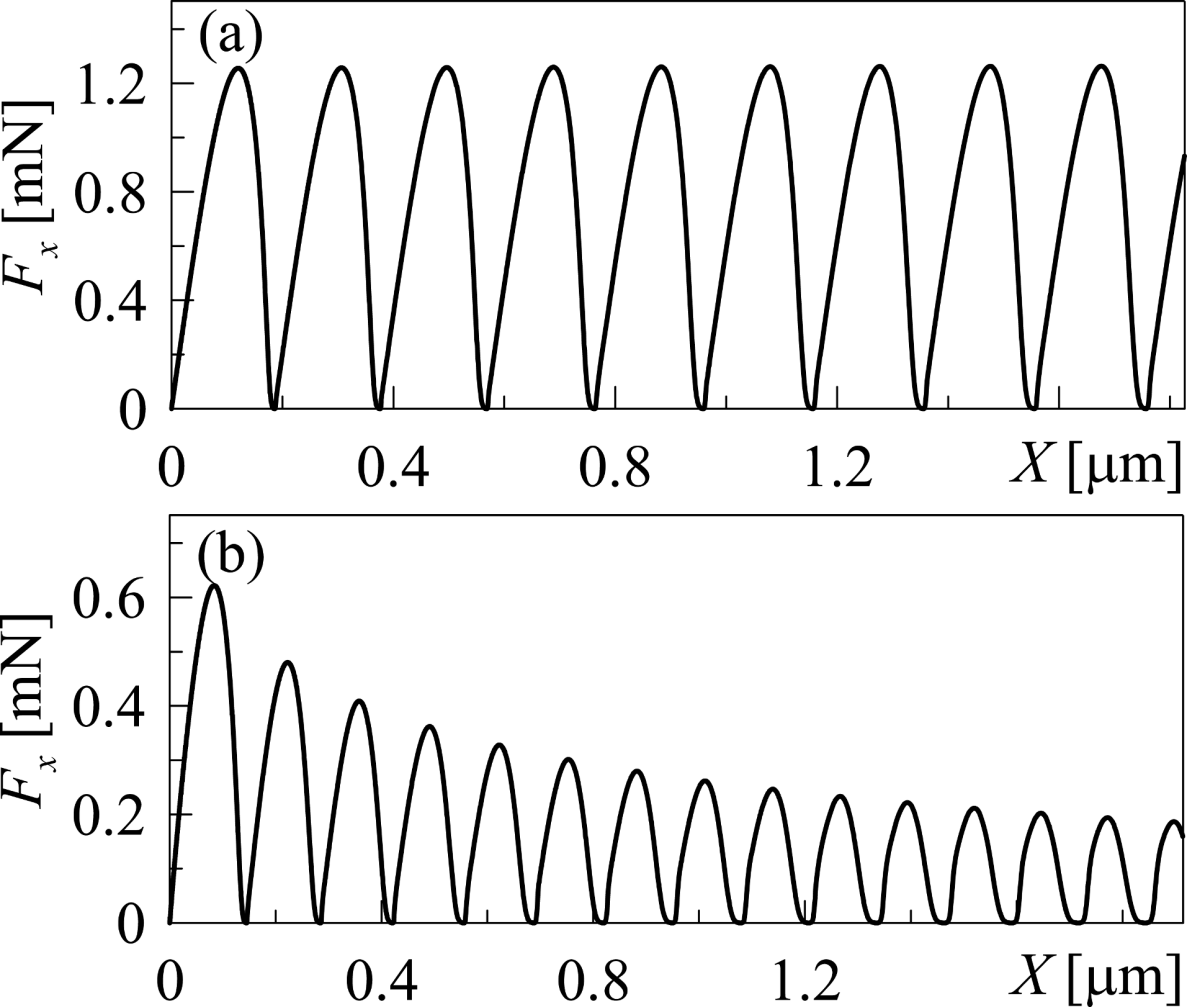
(a) Dependence of the friction force *F*_x_ on the stamp coordinate *X* (upper friction surface), corresponding to Figure 2a. (b) Dependence of the friction force *F*_x_ on the stamp coordinate *X*, corresponding to Figure 4a.

The dependence of the friction force *F*_x_ on the coordinate of the upper friction block *X* as shown in Figure 5 corresponds to the data from Figure 2a and Figure 4a.

As it can be seen from Figure 5, *F*_x_(*X*) is periodical (with damping oscillations in the second case, as the amplitude of the friction force decreases in time due to the heating of the lubricant). The presented dependencies have a regular form in contrast to the data in Figure 2 and Figure 4, where the frequency of the phase transitions increases with time. Different forms of the obtained dependencies can be explained as follows. Let us recall that in our simulations we introduced the constant velocity of the free edge of the spring *V*_0_ (see Figure 1). The velocity of the stamp center 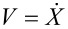 is calculated from Equation 10 and does not coincide with the velocity *V*_0_ mostly due to the presence of the friction force *F*_x_ (Equation 16). After the motion has begun, the tension of the spring and related growth of the elastic force causes the growth of the upper stamp velocity *V*. The lubricant melts in certain regions of the contact area where elastic stress, ε_el_(*r*), exceed a critical value, ε_el,c_ (Equation 4). As the velocity of the upper stamp *V* grows, the time needed for the elastic stress to reach the critical value ε_el,c_ decreases. Thus, the frequency of the phase transitions in Figure 2 and Figure 4 increases in time. However, strains (Equation 11) are determined by the magnitude of the displacement of the upper stamp over the bottom surface after another melting and subsequent solidification (when the lubricant solidifies after melting, strain is equal to zero for the subsequent growth according to Equation 11). Thus, the upper stamp, after another solidification of the lubricant, must pass approximately the same distance before the next melting, as is depicted in Figure 5. This situation confirms the assumption that the frequency of the phase transitions increases (as it is shown in Figure 2 and Figure 4) due to the increase in the strain rate.

## Conclusion

We have presented the dynamical simulation of the boundary friction between a cylindrically shaped stamp and a flat surface. Using the method of dimensionality reduction (MDR) we have studied the stick–slip friction mode that occurs in the tribological system under shear deformation. The MDR approach allowed us to describe the situation in which elastic stress, strain and order parameter are spatially distributed within contact area. The established stick–slip mode is characterized by continuous phase transitions between solid-like and liquid-like states of the lubricant, which were described as the second-order phase transitions between kinetic states of friction. Within the performed numerical simulation it is shown that an increase of the lubricant temperature leads to smaller amplitudes of the friction force, elastic stress and order parameter, while the frequency of phase transitions increases due to the presence of the spring. It is worth mentioning that the spatial distribution of elastic stress considered in the presented study will always occur in tribological systems with analogous geometrical shape of the contact area; thus, the developed approach can be an additional tool in various experimental investigations for contact problems of this type.
